# Complexity of Medication Regimens for Children With Neurological Impairment

**DOI:** 10.1001/jamanetworkopen.2021.22818

**Published:** 2021-08-26

**Authors:** James A. Feinstein, Hannah Friedman, Lucas E. Orth, Chris Feudtner, Allison Kempe, Sadaf Samay, Allison B. Blackmer

**Affiliations:** 1 Adult and Child Consortium for Health Outcomes Research and Delivery Science, University of Colorado and Children’s Hospital Colorado, Aurora; 2 Department of Pediatrics, University of Colorado, Aurora; 3 Pediatric Residency Program, University of Colorado, Aurora; 4 Skaggs School of Pharmacy and Pharmaceutical Sciences, University of Colorado, Aurora; 5 Division of General Pediatrics, Department of Pediatrics, The Children’s Hospital of Philadelphia, Philadelphia, Pennsylvania; 6 Research Informatics, Analytics Resource Center, Children’s Hospital Colorado, Aurora

## Abstract

**Importance:**

Parents of children with severe neurological impairment (SNI) manage complex medication regimens (CMRs) at home, and clinicians can help support parents and simplify CMRs.

**Objective:**

To measure the complexity and potentially modifiable aspects of CMRs using the Medication Regimen Complexity Index (MRCI) and to examine the association between MRCI scores and subsequent acute visits.

**Design, Setting, and Participants:**

This cross-sectional study was conducted between April 1, 2019, and December 31, 2020, at a single-center, large, hospital-based, complex care clinic. Participants were children with SNI aged 1 to 18 years and 5 or more prescribed medications.

**Exposure:**

Home medication regimen complexity was assessed using MRCI scores. The total MRCI score is composed of 3 subscores (dosage form, dose frequency, and specialized instructions).

**Main Outcomes and Measures:**

Patient-level counts of subscore characteristics and additional safety variables (total doses per day, high-alert medications, and potential drug-drug interactions) were analyzed by MRCI score groups (low, medium, and high score tertiles). Associations between MRCI score groups and acute visits were tested using Poisson regression, adjusted for age, complex chronic conditions, and recent health care use.

**Results:**

Of 123 patients, 73 (59.3%) were male with a median (interquartile range [IQR]) age of 9 (5-13) years. The median (IQR) MRCI scores were 46 (35-61 [range, 8-139]) overall, 29 (24-35) for the low MRCI group, 46 (42-50) for the medium MRCI group, and 69 (61-78) for the high MRCI group. The median (IQR) counts for the subscores were 6 (4-7) dosage forms per patient, 7 (5-9) dose frequencies per patient, and 5 (4-8) instructions per patient, with counts increasing significantly across higher MRCI groups. Similar trends occurred for total daily doses (median [IQR], 31 [20-45] doses), high-alert medications (median [IQR], 3 [1-5] medications), and potential drug-drug interactions (median [IQR], 3 [0-6] interactions). Incidence rate ratios of 30-day acute visits were 1.26 times greater (95% CI, 0.57-2.78) in the medium MRCI group vs the low MRCI group and 2.42 times greater (95% CI, 1.10-5.35) in the high MRCI group vs the low MRCI group.

**Conclusions and Relevance:**

Higher MRCI scores were associated with multiple dose frequencies, complicated by different dosage forms and instructions, and associated with subsequent acute visits. These findings suggest that clinical interventions to manage CMRs could target various aspects of these regimens, such as the simplification of dosing schedules.

## Key Points

**Question:**

How complex are the medication regimens for children with neurological impairment?

**Findings:**

In this cross-sectional study of 123 children with neurological impairment, empirically measured Medication Regimen Complexity Index scores were high; patients were prescribed a median of 31 total daily doses of medication, 7 different dose frequencies, 6 unique dosage forms, and 5 specialized instructions. Higher Medication Regimen Complexity Index scores were associated with increased subsequent acute health care utilization.

**Meaning:**

These findings suggest that clinical interventions to manage medication regimen complexity could target various aspects of these regimens, such as the simplification of dosing schedules.

## Teaser

This cross-sectional study uses the Medication Regimen Complexity Index to measure the complexity and potentially modifiable aspects of complex medication regimens for children with severe neurological impairment and examines the associations between the index scores and subsequent acute visits.

## Introduction

Complex medication regimens (CMRs)—usually defined as the use of 5 or more medications (polypharmacy) and the presence of multiple different medication dosing schedules—are common among children with medically complex conditions, including children with severe neurological impairment (SNI).^1,2,3^ Amid other competing care activities, parents and nonmedical caregivers of children with SNI must manage and administer CMRs in the home setting.^2^ In prior studies of medication safety,^4,5,6,7,8^ parents have reported concerns about medication administration, adherence, and adverse drug events (ADEs). The difficulty of administering CMRs can be affected by a variety of clinically modifiable medication-related factors, including the total number of medications, different dose frequencies, dosage forms (such as pills vs liquids), and specialized instructions, yet many pediatric polypharmacy studies still only measure basic medication counts.^9,10,11,12,13,14,15,16,17,18,19,20^ To guide clinicians’ efforts to simplify CMRs and to provide tailored support and education for parents who administer CMRs, clinicians need an easy and comprehensive way to identify and measure the various aspects of CMRs associated with medication complexity.^21,22^

The Medication Regimen Complexity Index (MRCI) is a validated tool that assesses medication regimen complexity in adult and geriatric populations with polypharmacy, and it has the potential to be extrapolated to the pediatric population.^16,19^ The MRCI score is intended to differentiate, for example, between a patient with lower complexity taking 10 medications, each as a single pill with once-daily dosing, and a patient with higher complexity taking 10 medications, but each with different dosage forms and requiring multiple doses per day. The total MRCI score is composed of 3 subscores calculated from commonly available elements of patients’ medication prescriptions: dosage form, dose frequency, and specialized instructions. MRCI scores have been used to identify adult patients most likely to benefit from pharmacist-led medication therapy management programs and have also shown value in estimating subsequent health care utilization and ADEs.^19,23^

In this study, we used the MRCI tool to identify, measure, and understand pediatric CMRs within a prototypical population of children with SNI and polypharmacy. We aimed to (1) measure the parent-facing complexity of CMRs using total MRCI scores and subscores; (2) describe medication-level characteristics corresponding to MRCI subscore domains; (3) describe patient-level characteristics of CMRs corresponding to MRCI subscore domains, as well as additional medication safety variables (eg, total daily doses, high-alert medications, and potential drug-drug interactions [PDDIs]), stratified by tertiles of total MRCI scores; and (4) test the hypothesis that higher total MRCI scores are associated with increased subsequent acute health care utilization, controlling for patient age, counts of complex chronic conditions (CCCs), and recent health care utilization.^23,24^

## Methods

### Study Design

This was a cross-sectional analysis of all patients enrolled in a larger study measuring symptoms and polypharmacy in children with SNI, as described in prior publications.^25,26^ We analyzed patients’ reconciled medication information captured in the electronic health record (EHR) during a routine primary care visit, as well as patients’ subsequent 30-day acute primary care, emergency, and inpatient health care utilization. The parent study was approved by the Colorado Multiple Institutional Review Board and registered with ClinicalTrials.gov (NCT03849066).^25,26^ This study follows the Strengthening the Reporting of Observational Studies in Epidemiology (STROBE) reporting guideline.^27^

### Identification, Consent, and Enrollment of Subjects

Between April 1, 2019, and December 31, 2020, we obtained parental written informed consent and enrolled and assessed English-speaking and Spanish-speaking children aged 1 to 18 years with SNI and polypharmacy (≥5 medications) who received primary care in a large, hospital-based special health care needs clinic. Consistent with previous definitions, children with SNI were defined as having neurological diagnoses expected to last 12 months or longer and resulting in systemic or multisystem physiological impairment requiring pediatric subspecialty care.^1,28^ The presence of SNI and counts of CCCs were identified using published classification systems based on *International Statistical Classification of Diseases, Tenth Revision, Clinical Modification* diagnosis codes.^1,29^ Parent-reported race/ethnicity was assessed based on prior studies demonstrating differences in prescribing patterns and medication use.^3^

### Medication Data

Prescription and over-the-counter medications were reconciled by the clinician at the time of the visit. All data elements necessary to calculate MRCI scores were available, including generic medication name, dose, dosage form, dose frequency, and additional specialized instructions. To reflect parent-facing medication complexity, we excluded clinic-administered or inpatient-administered medications (eg, vaccines or botulinum toxin injections). Medication classes were assigned using the Anatomic Therapeutic Chemical classification system.^30^

### MRCI Score and Subscores

MRCI scores were calculated automatically from EHR data using the MRCI tool, scoring instructions, and examples that are publicly available.^31^ Conceptually, the total MRCI score for a CMR is the sum of 3 weighted subscores (dosage form, dose frequency, and specialized instructions), with increasing weights corresponding to the difficulty of administration.^16,19^ Each weighted dosage form score (eg, tablet = 1, liquid = 2) was counted once per CMR. Each weighted dose frequency score (eg, once daily = 1, twice daily = 2, every 12 hours = 2.5) was counted once per medication. Each weighted specialized instruction score (eg, take multiple units = 1, taper or increase dose = 2) was counted once per medication. In the simplest scenario, a tablet administered once daily has a dosage form subscore of 1, dose frequency subscore of 1, and an instruction subscore of 0, for a total MRCI score of 2. The total MRCI score has no upper limit because it is dependent on the total number of medications, and higher MRCI scores indicate more-complex regimens. Because meaningful MRCI score thresholds have not yet been established within the pediatric population, we classified MRCI scores by tertiles into low, medium, and high categories.

### Additional Medication Administration and Safety Characteristics of CMR

We assessed additional medication safety–related variables, including total daily doses, counts of high-alert medications, and counts of PDDIs.^2,32^ Total daily dose counts were parameterized as minimum counts, based on scheduled medications, and maximum counts, based on scheduled medications plus as-needed (PRN) medications. High-alert medications have the potential to cause substantial patient harm when used in error and were identified using published lists from the Institute for Safe Medication Practices.^33^ Finally, PDDIs were identified using DrugBank’s interaction database.^34^ Interactions of moderate severity (ie, monitor and/or modify concomitant use) and major severity (ie, avoid concomitant use) were reported.^34^

### Clinical and Healthcare Utilization Variables

Additional analytical variables were extracted from the patient’s EHR. Variables included patient and parent demographic information; annual and visit *International Statistical Classification of Diseases, Tenth Revision, Clinical Modification* diagnoses; and annual and subsequent 30-day acute health care utilization (acute primary care, emergency, and inpatient visits).

### Statistical Analysis

Descriptive statistics and distributional graphs were used to describe the study population and the associated MRCI scores. Bivariate comparisons between patient characteristics or health care utilization and MRCI scores were evaluated using 1-way analysis of variance tests. Box plots were used to visualize the corresponding characteristics of CMR, stratified by MRCI score tertiles. Nonparametric tests of trend were used to assess for differences in median counts of characteristics of CMR across MRCI score categories. Multivariable Poisson regression was used to test the hypothesis that higher MRCI scores were associated with increased subsequent 30-day acute health care utilization, adjusting for variables known to be associated with acute health care utilization (age, number of CCCs, and acute health care utilization in the prior 30 days).^24^ Analyses were conducted using Stata statistical software version 16.1 (StataCorp), and significance was set at a 2-tailed *P* < .05.

## Results

### Demographic and Clinical Characteristics of Study Patients and Parent Caregivers

Of the 123 patients with SNI included in the analysis, most were male (73 patients [59.3%]) and White (94 patients [76.4%]), with a median (interquartile range [IQR]) age of 9 (5-13) years and a high prevalence of 3 or more CCCs (74 patients [60.1%]) (Table 1). Patients frequently had 10 or more active medications (98 patients [79.7%]) directly before the clinical visit. Prior health care utilization was high, with numerous outpatient medical visits (51 patients [41.5%] with ≥20 visits annually), emergency visits (50 patients [40.7%] with ≥2 visits annually), and inpatient stays (61 patients [49.6%] with ≥2 stays annually). The parent caregivers for the study patients were primarily between 30 and 50 years old (95 caregivers [77.2%]), female (106 caregivers [86.2%]), non-Hispanic or non-Latino (93 caregivers [75.6%]), White (97 caregivers [78.9%]), and had some college education (99 caregivers [80.5%]). Sixty-one patients (49.6%) had 1 day or more per week of nurse-provided care.

**Table 1.** Demographic and Clinical Characteristics of Children With Severe Neurological Impairment by MRCI Score

Characteristic	Patients, No. (%) (N = 123)	MRCI score, mean (95% CI)	*P* value^a^
Patient age at visit, y			
1-4	25 (20.3)	44.8 (39.0-50.6)	.20
5-8	34 (27.6)	54.9 (49.2-60.6)	
9-12	35 (28.5)	49.1 (40.5-57.7)	
13-17	29 (23.6)	45.2 (37.1-53.3)	
Patient sex			
Male	73 (59.3)	49.6 (44.3-54.9)	.66
Female	50 (40.7)	47.9 (43.1-52.7)	
Patient race			
American Indian or Alaska Native	1 (0.8)	69.0 (NA)	.91
Black or African American	5 (4.1)	48.1 (33.6-62.6)	
White	94 (76.4)	49.0 (44.5-53.5)	
>1 Race	17 (13.8)	48.7 (41.6-55.8)	
Not specified	6 (4.9)	45.6 (29.4-61.8)	
Patient ethnicity			
Not Hispanic or Latino	94 (76.4)	49.1 (44.5-53.7)	.88
Hispanic or Latino	29 (23.6)	48.4 (42.9-53.9)	
Complex chronic conditions, No.			
1-2	49 (39.8)	42.1 (37.1-47.1)	<.001
3-4	55 (44.7)	49.5 (44.2-54.8)	
≥5	19 (15.4)	64.8 (54.5-75.1)	
Previsit prescriptions, No.			
5-9	25 (20.3)	28.9 (24.8-33.0)	<.001
10-14	44 (35.8)	41.0 (37.1-44.9)	
≥15	54 (43.9)	64.7 (59.8-69.6)	
Outpatient visits in past year, No.			
1-9	30 (24.4)	38.2 (32.6-43.8)	.003
10-19	42 (34.1)	49.3 (43.7-54.9)	
20-29	27 (22.0)	51.4 (44.8-58.0)	
≥30	24 (19.5)	59.0 (47.6-70.4)	
Emergency department visits in past year, No.			
0	53 (43.1)	46.6 (41.5-51.7)	.10
1	20 (16.3)	45.7 (38.6-52.8)	
2	22 (17.9)	46.4 (38.6-54.2)	
≥3	28 (22.8)	57.6 (47.5-67.7)	
Inpatient visits in past year, No.			
0	34 (27.6)	38.9 (33.8-44.0)	<.001
1	28 (22.8)	43.8 (37.0-50.6)	
2	24 (19.5)	56.8 (48.3-65.3)	
≥3	37 (30.1)	56.9 (49.6-64.2)	
Parent education			
Some high school	5 (4.1)	49.2 (28.2-70.2)	.84
High school graduate	18 (14.6)	45.0 (38.0-52.0)	
Some college or technical school	48 (39.0)	50.3 (43.4-57.2)	
College graduate	51 (41.5)	49.5 (44.2-54.8)	
Not specified	1 (0.8)	23.5 (NA)	
Nurse-provided care, No. of days/week			
0	59 (48.0)	45.8 (40.5-51.1)	.06
1-3	22 (17.9)	47.9 (37.7-58.1)	
4-7	39 (31.7)	55.5 (49.8-61.2)	
Not specified	3 (2.4)	31.7 (19.4-44.0)	

Abbreviations: MRCI, Medication Regimen Complexity Index; NA, not applicable.

^a^ Calculated with 1-way analysis of variance.

### MRCI Scores and Subscores

**Figure 1.**
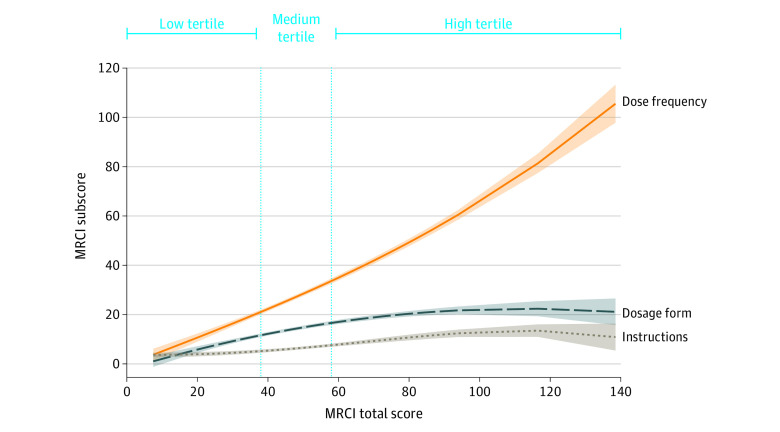
Distribution of Medication Regimen Complexity Index (MRCI) Subscores by Total MRCI Scores in Children With Severe Neurological Impairment This graph displays fractional polynomial plots of MRCI mean subscores associated with total MRCI scores. The vertical reference lines display thresholds for the low, medium, and high MRCI total score tertiles. Shaded areas denote 95% CIs.

The median (IQR) MRCI scores for patients were 46 (35-61 [range, 8-139]) overall, 29 (24-35) for the low MRCI group, 46 (42-50) for the medium MRCI group, and 69 (61-78) for the high MRCI group (Figure 1). The median (IQR) MRCI subscores were 14 (9-18) for dosage form, 25 (19-39) for dose frequency, and 6 (4-9) for additional instructions. Each MRCI subscore contributed differentially to the MRCI total score (Figure 1), with the dose frequency subscore contributing most to medication complexity, particularly at higher total MRCI scores. Total MRCI scores varied significantly by certain demographic characteristics, including number of CCCs (mean MRCI scores, 42.1 [95% CI, 37.1-47.1] for 1-2 CCCs, 49.5 [95% CI, 44.2-54.8] for 3-4 CCCs, and 64.8 [95% CI, 54.5-75.1] for ≥5 CCCs; *P* < .001) and total number of medications (mean MRCI scores, 28.9 [95% CI, 24.8-33.0] for 5-9 prescriptions, 41.0 [95% CI, 37.1-44.9] for 10-14 prescriptions, and 64.7 [95% CI, 59.8-69.6] for ≥15 prescriptions; *P* < .001). MRCI scores also differed significantly by preceding health care use, including outpatient visits in past year (mean MRCI score, 38.2 [95% CI, 32.6-43.8] for 1-9 visits, 49.3 [95% CI, 43.7-54.9] for 10-19 visits, 51.4 [95% CI, 44.8-58.0] for 20-29 visits, and 59.0 [95% CI, 47.6-70.4] for ≥30 visits; *P* = .003) and inpatient visits in the past year (mean MRCI score, 38.9 [95% CI, 33.8-44.0] for 0 visits, 43.8 [95% CI, 37.0-50.6] for 1 visit, 56.8 [95% CI, 48.3-65.3] for 2 visits, and 56.9 [95% CI, 49.6-64.2] for ≥3 visits; *P* < .001) (Table 1). MRCI scores did not differ significantly by age, sex, race, ethnicity, annual emergency visits, parent education level, or days of or nurse-provided care.

### Medication-Level Characteristics of CMR by MRCI Subscore Domains

**Figure 2.**
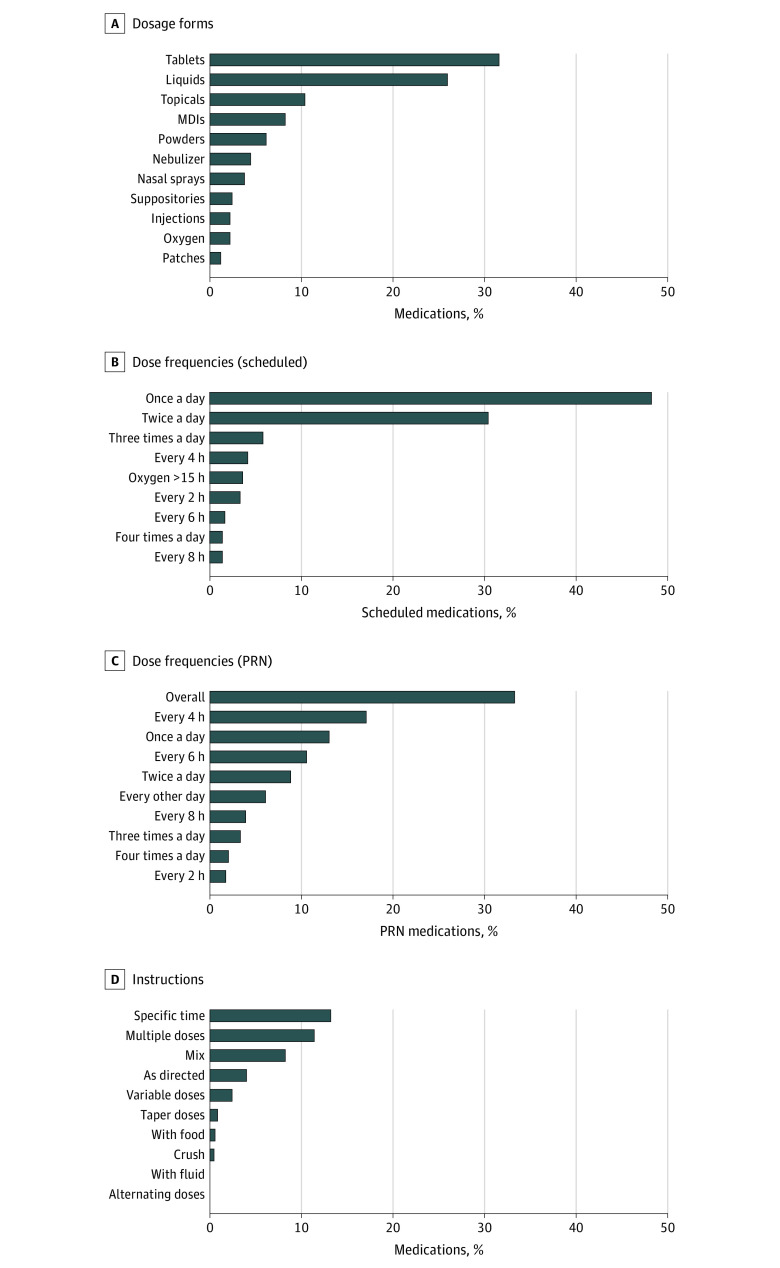
Medication-Level Characteristics of Complex Medication Regimens Related to Medication Regimen Complexity Index (MRCI) Subscores in Children With Severe Neurological Impairment Graphs show the percentage distribution of dosage forms among all medications (A), the percentage distribution of dose frequencies among all scheduled medications (B), the percentage distribution of dose frequencies among all as-needed (PRN) medications (C), and the percentage distribution of additional instructions among all medications (D). MDI indicates meter dose inhaler.

Patients were prescribed a total of 1772 prescriptions, including 210 distinct generic medications (eTable in the Supplement). Regarding dose forms, capsules and tablets accounted for 31.6% (560 of 1772 medications) and liquids accounted for 26.0% (460 of 1772 medications) of medications (Figure 2A). More than one-half (560 of 1082 medications [51.8%]) of all scheduled individual medications required 2 or more doses per day (Figure 2B), most frequently antiepileptics (15.1% of scheduled medications with ≥2 doses per day), asthma medications (14.3%), antacids (8.9%), systemic steroids (6.5%), and anxiolytics (4.4%). Similarly, 47.5% (328 of 690 medications) of all PRN medications had recommended dose frequencies of 2 or more doses per day, whereas 33.3% (230 of 690 medications) of PRN medications did not have a specified dose frequency (Figure 2C). Finally, 34.8% (616 of 1772 medications) of all medications had 1 or more associated specialized instruction, most frequently indicating specific times of administration (234 of 1772 medications [13.2%]), use of multiple units (202 of 1772 medications [11.4%]), and medication mixing or dissolving instructions (146 of 1772 medications [8.2%]) (Figure 2D).

### Patient-Level Characteristics of CMR by MRCI Total Scores

**Figure 3.**
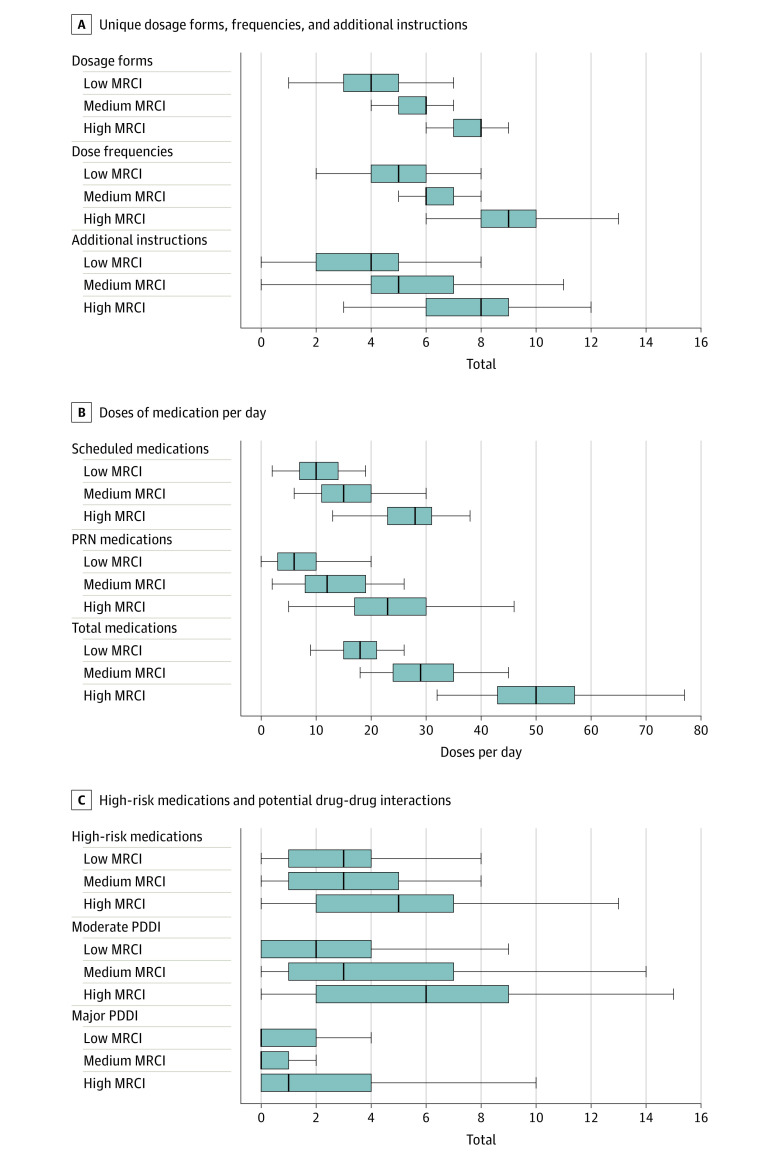
Characteristics of Complex Medication Regimens at the Patient Level by Medication Regimen Complexity Index (MRCI) Tertile in Children With Severe Neurological Impairment In the box plots, lines within boxes denote medians, ends of boxes denote interquartile ranges, and error bars denote the lower and upper adjacent values. Panel A displays counts for unique dosage forms per patient, dose frequencies per patient, and additional instructions per patient. Panel B displays counts for scheduled medication doses per patient per day excluding as-needed (PRN) medications, PRN medications per patient per day, and total doses per patient per day. Panel C displays counts for high-risk medications per patient, and moderate and major potential drug-drug interactions per patient. All comparisons across MRCI score groups were significant (*P* < .05, nonparametric tests of trend).

Overall, median (IQR) counts were 6 (4-7) different dosage forms per day, 7 (5-9) dose frequencies per day, and 5 (4-8) additional instructions (Figure 3A). Similarly, median (IQR) dose counts were 16 (11-26) total scheduled doses of medication per day, 12 (7-20) PRN medication doses per day, and 31 (20-45) total doses of medication per day (Figure 3B). Finally, median (IQR) counts were 3 (1-5) high-risk medications per participant, 3 (0-6) moderate PDDIs, and 0 (0-3) major PDDIs (Figure 3C). For each of the characteristics of the CMR, the median counts increased significantly across higher MRCI score groups (Figure 3). For example, the median (IQR) total doses per day were 18 (15-21) for the low MRCI group, 29 (24-35) for the medium MRCI group, and 50 (43-57) for the high MRCI group; the increase in median total daily doses across MRCI score groups was significant (*P* < .05, nonparametric test of trend).

### Association Between MRCI Scores and 30-Day Acute Healthcare Utilization

Unadjusted 30-day incidence rate ratios of acute health care utilization were 0.3 visit per person-month in the low MRCI group, 0.4 visit per person-month in the medium MRCI group, and 0.7 visit per person-month in the high MRCI group (Table 2). After adjustments for age, number of CCCs, and preceding 30-day acute health care utilization, the incidence rate ratio of subsequent 30-day acute visits was 1.26 times greater (95% CI, 0.57-2.78) in the medium group and 2.42 times greater (95% CI, 1.10-5.35) in the high group (Table 2).

**Table 2.** Association Between MRCI Total Scores and Subsequent 30-Day Acute Healthcare Utilization in Children With Severe Neurological Impairment

Variable	Participants, No.	Acute visits, No.	IRR (95% CI)	
			Unadjusted	Adjusted^a^
Patient age, y				
1-4	25	20	2.80 (1.31-5.98)	2.61 (1.16-5.88)
5-8	34	15	1.54 (0.69-3.44)	1.30 (0.58-2.93)
9-12	35	10	1 [Reference]	1 [Reference]
13-17	29	11	1.33 (0.56-3.13)	1.36 (0.57-3.25)
Complex chronic conditions, No.				
1-2	49	16	1 [Reference]	1 [Reference]
3-4	55	25	1.39 (0.74-2.61)	1.05 (0.55-2.01)
≥5	19	15	2.42 (1.20-4.89)	1.04 (0.45-2.38)
MRCI score category				
Low	41	11	1 [Reference]	1 [Reference]
Medium	41	15	1.36 (0.63-2.97)	1.26 (0.57-2.78)
High	41	30	2.72 (1.37-5.44)	2.42 (1.10-5.35)
30-d Acute visits (previsit)	123	56	1.47 (1.21-1.77)	1.23 (0.99-1.53)

Abbreviations: IRR, incidence rate ratio; MRCI, Medication Regimen Complexity Index.

^a^ Adjusted for age, number of complex chronic conditions, and preceding 30-day acute health care utilization.

## Discussion

In this cross-sectional study, children with SNI and polypharmacy displayed a broad range of medication regimen complexity, with MRCI scores ranging from 8 to 139 and a median 46. During nonacute, routine clinical visits, children with SNI had substantially higher median MRCI scores than those reported in other analogous populations. In previous studies,^23,35,36,37,38,39,40,41^ adults with autism spectrum disorder had mean MRCI scores of 14.6 (range, 0-89), and geriatric patients with complex polypharmacy had mean scores ranging from 9.9 in the primary care setting to 30.3 at hospital discharge, with scores of 22 or higher associated with unplanned 30-day readmissions. In the present study, 26.0% of all medications were liquids (measuring liquids contributes to both complexity and higher risk for dosing errors), and 51.8% of all scheduled individual medications required 2 or more doses per day.^8,12,42^ Multiple different dose frequencies per patient were associated with higher MRCI scores, which for those in the high MRCI group, manifested as a median of 50 total daily doses per day. Additional characteristics of CMR directly affecting patient safety, including counts of high-alert medications and PDDIs, were also associated with higher MRCI scores. Finally, consistent with geriatric literature,^17,19,23,43,44,45^ higher MRCI scores in the current study were associated with increased subsequent 30-day acute health care utilization.

These findings have several implications for the identification, clinical management, and ongoing monitoring of children with the most complex medication regimens. First, MRCI scores are easily calculated and can optimize the identification of patients who would benefit from clinical management, ideally coordinated by primary care physicians and pharmacists. Even within an already complex population of children with polypharmacy, correct identification of children with the most complex medication regimens is important for efficiency and safety, especially when clinical pediatric pharmacist support is limited.^46,47^ Currently, no other standard measures of pediatric medication complexity are routinely used to trigger CMR management. Evidence-based MRCI thresholds could flag a patient’s EHR in real time to indicate a need for pharmacist review and medication therapy management interventions to simplify the CMR.^11,48^ Within health care systems, quantifying the volume of patients who would benefit from pharmacist-led interventions would help substantiate the need and value of providing dedicated pharmacist support within complex care programs and medical homes.^20,49^

Next, primary care clinicians and pharmacists could use MRCI scores during clinical encounters to direct efforts to deprescribe and simplify CMR. On the basis of the MRCI subscores in this population of children with SNI, clinicians should first focus on ways to reduce total doses per day, through reductions in dose frequency or numbers of medications. In our experience, dose frequency reductions may be achieved by evaluating regimens for substitution with a therapeutic equivalent allowing for fewer doses per day, or dosage form optimization to change from immediate-release preparations to less-frequent extended-release preparations, with the caveat that extended-release products cannot be crushed for enteral administration. Further reductions in total number of medications can be achieved by evaluating regimens for removal of therapeutic duplications; opportunities to wean long-term medications used for well-controlled stable disease states, especially for legacy medications started years ago, where therapy may no longer be necessary; and outgrown doses, used as an opportunity to determine whether the drug is still needed, rather than simply increasing to a weight-appropriate dose.

Reductions in doses per day may not always be possible, so pharmacists may support parents through additional tailored solutions and specific education interventions. Pill packs or pill boxes can be used to group medications that can be administered together at a common time, effectively reducing administration burden.^50^ When MRCI subscores reveal complexity resulting from multiple dosage forms or specialized instructions, pharmacist-led demonstrations and practice opportunities may improve parental confidence administering medications.^2,5,51^ A subset of liquid dosage forms require extemporaneous compounding; risks associated with compounded medications may be reduced by switching to commercially available products, such as tablets or capsules that can be crushed or opened. Standardization of specialized instructions, made available in multiple languages and with diagrams, could also reduce variability in clinicians’ free-text prescription instructions and improve parental understanding and confidence.^8,12,42,51^

Finally, for children with a CMR, studying the downstream clinical impact of interventions that reduce medication regimen complexity is difficult because no standard method is consistently used to measure pediatric CMRs as either as predictor or outcome variables.^20,52^ Moreover, in patient populations where medication changes occur frequently or simultaneously, without a composite measure of medication regimen complexity, determining the effect of a single medication change amid a milieu of multiple medication changes is daunting. Rather, the composite MRCI score captures changes resulting from interventions affecting dosage forms, dose frequencies, and additional instructions, and MRCI scores may prove useful in tracking medication regimen complexity over time. Interventions to simplify and optimize CMRs should be evaluated by longitudinally analyzing the association between MRCI scores and important clinical outcomes, such as patient-reported or parent-reported symptoms, exposure to PDDIs or high-alert medications, evidence of ADEs, or acute health care utilization.^10,23^ For example, our finding that increased 30-day acute care utilization was associated with the highest MRCI score group warrants further investigation. Given the confounding between complexity of the patient’s medical conditions and the CMR, we should not assume that reductions in MRCI scores would reduce acute health care utilization. Higher MRCI scores may instead reflect severity of illness, rather than contributing directly to ADEs. Further evaluations of causality should use a consistent measure of medication regimen complexity, such as MRCI scores, to reduce methodological heterogeneity.^20,52^

### Limitations

Our study must be considered in the context of 4 limitations. First, this study was conducted at a single center in a primarily White, non-Hispanic, and English-speaking population with a focused and, thus, limited (albeit typical) set of severe neurological conditions. The utility of MRCI scores should be tested in diverse populations of children with medical complexity and different underlying disease processes. Second, the chosen thresholds for MRCI score groups were based on score percentiles rather than absolute cut points because no prior pediatric MRCI benchmarks were available. Until MRCI score data are generated for broader pediatric populations with different disease processes, the thresholds used in this study should not be used as absolute thresholds. Third, MRCI scores do not capture all unique pediatric-specific aspects of a medication regimen that may add significant complexity, such as compounded medications, use of enteral feeding tubes, or number of unique caregivers administering medications. Because these important CMR aspects often occur in the care of children, pediatric-specific modification and validation of the MRCI tool may be valuable. Finally, on the basis of previous reports in which MRCI scores were associated with acute care utilization due to ADEs, we used acute health care utilization as a proxy for medication-related issues (after adjusting for other markers of medical complexity). We did not, however, perform a causality assessment of ADEs.^53^

## Conclusions

In this cross-sectional study, MRCI scores were easily calculated from readily available medication data in the EHR and provided meaningful and actionable information. As MRCI scores increased, parents administered many more doses of medications per day, complicated by different dosage forms and specialized instructions. Higher MRCI scores were associated with increased subsequent acute health care utilization. These findings suggest that clinical interventions to manage CMRs could target various aspects of these regimens, such as the simplification of dosing schedules.

## Notes

Supplement. **eTable.** Medications Used by MRCI Tertile in Children With Severe Neurological Impairment
