# Supplementary material for: Complexity of Medication Regimens for Children With Neurological Impairment
Source: JAMA Netw Open. 2021 Aug 26;4(8):e2122818. doi: 10.1001/jamanetworkopen.2021.22818 (PMC8391103; doi:10.1001/jamanetworkopen.2021.22818)
Supplement: Supplement. — eTable. Medications Used by MRCI Tertile in Children With Severe Neurological Impairment [file jamanetwopen-e2122818-s001.pdf]

Supplemental Online Content

Feinstein JA, Friedman H, Orth LE, et al. Complexity of medication regimens for children with neurological impairment. *JAMA Netw Open.* 2021;4(8):e2122818. doi:10.1001/jamanetworkopen.2021.22818

eTable. Medications Used by MRCI Tertile in Children With Severe Neurological Impairment

This supplemental material has been provided by the authors to give readers additional information about their work.

© 2021 Feinstein JA et al. *JAMA Network Open.*

eTable. Medications Used by MRCI Tertile in Children With Severe Neurological Impairment

Generic Name	% of Patients in Low MRCI Tertile Using Medication (N=41)	% of Patients in Medium MRCI Tertile Using Medication (N=41)	% of Patients in High MRCI Tertile Using Medication (N=41)
acetazolamide	4.9	4.9	0.0
acetylsalicylic acid	4.9	4.9	7.3
alpha-d-galactosidase	0.0	0.0	2.4
amantadine	2.4	0.0	0.0
amfetamine	2.4	4.9	0.0
aminocaproic acid	0.0	7.3	0.0
amitriptyline	2.4	4.9	4.9
amlodipine	0.0	2.4	0.0
amoxicillin	2.4	0.0	7.3
amoxicillin and beta-lactamase	0.0	4.9	0.0
anakinra	0.0	0.0	2.4
aripiprazole	0.0	2.4	2.4
ascorbic acid	0.0	7.3	7.3
atropine	0.0	0.0	2.4
azelastine	7.3	4.9	0.0
azithromycin	4.9	4.9	4.9
bacitracin	4.9	0.0	2.4
baclofen	14.6	19.5	14.6
beclometasone	7.3	2.4	0.0
benzethonium	0.0	2.4	0.0
benzocaine	0.0	2.4	0.0
benzonatate	0.0	0.0	2.4
betamethasone	0.0	2.4	0.0
bethanechol	2.4	0.0	0.0
bioflavonoids	2.4	0.0	0.0
bisacodyl	2.4	4.9	4.9
bosentan	0.0	0.0	2.4
budesonide	7.3	4.9	7.3
buprenorphine	2.4	0.0	0.0
calcium compounds	12.2	2.4	4.9
cannabidiol	2.4	2.4	0.0
captopril	0.0	4.9	0.0
carbidopa	0.0	2.4	0.0
carbidopa-levodopa	0.0	2.4	0.0
cefalexin	2.4	0.0	0.0

© 2021 Feinstein JA et al. *JAMA Network Open.*

cefdinir	0.0	2.4	0.0
ceftriaxone	0.0	0.0	2.4
celecoxib	0.0	2.4	0.0
cetirizine	39.0	26.8	31.7
chlorhexidine	0.0	0.0	2.4
chlorothiazide	0.0	7.3	0.0
cholestyramine	0.0	0.0	4.9
ciclesonide	0.0	2.4	2.4
citalopram	4.9	2.4	2.4
clindamycin	4.9	7.3	4.9
clobazam	17.1	12.2	17.1
clobetasol	0.0	4.9	0.0
clonazepam	12.2	12.2	19.5
clonidine	24.4	9.8	19.5
clotrimazole	0.0	4.9	2.4
colecalciferol	14.6	24.4	24.4
colistimethate	0.0	0.0	4.9
crisaborole	0.0	2.4	0.0
cyclobenzaprine	0.0	4.9	0.0
cyproheptadine	2.4	12.2	19.5
dantrolene	0.0	0.0	2.4
desmopressin	2.4	2.4	2.4
desonide	14.6	14.6	14.6
dexamethasone	2.4	0.0	2.4
dexmethylphenidate	7.3	0.0	0.0
diazepam	39.0	46.3	48.8
diclofenac	7.3	4.9	7.3
dicyclomine	0.0	7.3	2.4
diethyltoluamide	2.4	0.0	0.0
dihydroergotamine	0.0	2.4	0.0
dimethicone	0.0	2.4	0.0
dimetindene	0.0	2.4	0.0
diphenhydramine	7.3	9.8	14.6
docusate sodium	7.3	4.9	12.2
dornase alfa	4.9	2.4	0.0
doxepin	2.4	0.0	0.0
duloxetine	2.4	0.0	0.0
enoxaparin	2.4	0.0	2.4
epinephrine	9.8	12.2	9.8
ergocalciferol	0.0	2.4	0.0
erythromycin	7.3	0.0	7.3

© 2021 Feinstein JA et al. *JAMA Network Open.*

escitalopram	4.9	0.0	0.0
famotidine	4.9	9.8	2.4
fexofenadine	4.9	2.4	4.9
fludrocortisone	4.9	0.0	4.9
fluocinolone acetonide	7.3	4.9	4.9
fluocinonide	2.4	12.2	7.3
fluoxetine	2.4	0.0	2.4
fluticasone	70.7	68.3	63.4
folic acid	0.0	2.4	0.0
furosemide	0.0	2.4	7.3
gabapentin	14.6	9.8	9.8
gentamicin	0.0	2.4	0.0
ginger-fennel	0.0	0.0	2.4
glycopyrronium bromide	0.0	7.3	14.6
guaifenesin	2.4	0.0	0.0
guanfacine	4.9	7.3	4.9
hydrochlorothiazide	0.0	4.9	0.0
hydrocortisone	22.0	22.0	19.5
hydroxychloroquine	2.4	0.0	0.0
hydroxyzine	9.8	2.4	12.2
hyoscyamine	2.4	0.0	2.4
ibuprofen	22.0	22.0	9.8
imipramine	2.4	0.0	0.0
ipratropium bromide	2.4	7.3	31.7
iron bivalent	34.1	22.0	24.4
isotretinoin	0.0	0.0	2.4
ketoconazole	4.9	4.9	0.0
ketotifen	0.0	4.9	0.0
lacosamide	7.3	4.9	2.4
lactulose	4.9	2.4	2.4
lamotrigine	9.8	4.9	0.0
lansoprazole	9.8	7.3	22.0
levetiracetam	19.5	22.0	9.8
levocarnitine	17.1	4.9	4.9
levocetirizine	2.4	2.4	0.0
levonorgestrel	0.0	2.4	0.0
levothyroxine sodium	9.8	9.8	2.4
lidocaine	9.8	7.3	12.2
lidocaine-prilocaine	0.0	4.9	4.9
lisinopril	0.0	2.4	0.0
loperamide	2.4	0.0	2.4

© 2021 Feinstein JA et al. *JAMA Network Open.*

loratadine	4.9	7.3	4.9
lorazepam	2.4	7.3	4.9
macrogol	75.6	56.1	46.3
magnesium compounds	14.6	26.8	12.2
melatonin	46.3	22.0	26.8
meloxicam	2.4	0.0	2.4
menthol-zinc	14.6	9.8	9.8
methylphenidate	9.8	2.4	0.0
methylprednisolone	0.0	0.0	7.3
metronidazole	0.0	2.4	2.4
miconazole	7.3	2.4	9.8
midazolam	34.1	22.0	22.0
midodrine	0.0	0.0	2.4
mirtazapine	2.4	0.0	2.4
mometasone	17.1	14.6	17.1
montelukast	29.3	31.7	24.4
multienzymes (lipase, protease etc.)	4.9	0.0	2.4
multivitamins, plain	56.1	36.6	24.4
mupirocin	4.9	7.3	9.8
mycophenolic acid	0.0	2.4	0.0
naproxen	4.9	4.9	0.0
neomycin-bacitracin-polymyxin	2.4	2.4	4.9
nystatin	14.6	24.4	31.7
ofloxacin	2.4	0.0	4.9
olanzapine	2.4	0.0	0.0
olopatadine	2.4	17.1	12.2
omeprazole	22.0	14.6	9.8
ondansetron	26.8	26.8	29.3
oxcarbazepine	4.9	4.9	2.4
oxybutynin	4.9	2.4	2.4
oxycodone	0.0	7.3	7.3
oxygen	26.8	26.8	41.5
oxymetazoline	2.4	2.4	0.0
palivizumab	2.4	2.4	0.0
paracetamol	29.3	43.9	19.5
pentamidine	0.0	0.0	2.4
phenazopyridine	0.0	2.4	0.0
phenobarbital	0.0	9.8	4.9
phenylephrine-bromphen-dm	2.4	0.0	0.0
potassium	9.8	4.9	7.3
prednisolone	0.0	7.3	9.8

© 2021 Feinstein JA et al. *JAMA Network Open.*

prednisone	0.0	4.9	0.0
pregabalin	2.4	0.0	0.0
prochlorperazine	2.4	7.3	2.4
progestogens and estrogens	9.8	7.3	7.3
promethazine	0.0	2.4	4.9
propranolol	4.9	0.0	0.0
pyridoxine (vit b6)	2.4	4.9	2.4
quetiapine	7.3	2.4	4.9
ramelteon	0.0	4.9	0.0
ranitidine	31.7	19.5	14.6
risperidone	12.2	0.0	2.4
rivaroxaban	0.0	2.4	0.0
rizatriptan	7.3	7.3	2.4
rufinamide	4.9	4.9	0.0
salbutamol	97.6	90.2	100.0
scopolamine	2.4	4.9	4.9
senna glycosides	17.1	19.5	17.1
sertraline	9.8	4.9	0.0
sildenafil	7.3	12.2	2.4
sirolimus	0.0	2.4	0.0
sodium bicarbonate	0.0	2.4	0.0
sodium chloride, hypertonic	14.6	14.6	29.3
sodium citrate	4.9	2.4	2.4
somatropin	4.9	7.3	2.4
spironolactone	0.0	0.0	2.4
stiripentol	0.0	2.4	0.0
sucralfate	2.4	0.0	2.4
sulfasalazine	0.0	2.4	0.0
sumatriptan	0.0	7.3	2.4
tacrolimus	0.0	7.3	0.0
tadalafil	2.4	2.4	2.4
testosterone	2.4	0.0	0.0
thiamine (vit b1)	2.4	0.0	0.0
tiotropium bromide	0.0	2.4	0.0
tizanidine	2.4	4.9	0.0
tobramycin	0.0	0.0	2.4
topiramate	7.3	7.3	7.3
tramadol	4.9	2.4	0.0
tranexamic acid	0.0	2.4	0.0
trazodone	14.6	12.2	9.8
tretinoin	4.9	0.0	0.0

© 2021 Feinstein JA et al. *JAMA Network Open.*

triamcinolone	34.1	26.8	24.4
trihexyphenidyl	0.0	0.0	2.4
valproic acid	7.3	7.3	2.4
venlafaxine	0.0	2.4	0.0
vigabatrin	2.4	0.0	2.4
vitamin	2.4	0.0	0.0
vitamin k	0.0	2.4	0.0
warfarin	0.0	4.9	0.0
zinc	2.4	2.4	14.6
zonisamide	4.9	4.9	4.9

© 2021 Feinstein JA et al. *JAMA Network Open.*
